# False occurrences of functional motifs in protein sequences highlight evolutionary constraints

**DOI:** 10.1186/1471-2105-8-68

**Published:** 2007-03-01

**Authors:** Allegra Via, Pier Federico Gherardini, Enrico Ferraro, Gabriele Ausiello, Gianpaolo Scalia Tomba, Manuela Helmer-Citterich

**Affiliations:** 1 Centro di Bioinformatica Molecolare, Department of Biology, University of Rome Tor Vergata, Roma; 2 Department of Mathematics, University of Rome Tor Vergata, Roma

## Abstract

**Background:**

False occurrences of functional motifs in protein sequences can be considered as random events due solely to the sequence composition of a proteome. Here we use a numerical approach to investigate the random appearance of functional motifs with the aim of addressing biological questions such as: How are organisms protected from undesirable occurrences of motifs otherwise selected for their functionality? Has the random appearance of functional motifs in protein sequences been affected during evolution?

**Results:**

Here we analyse the occurrence of functional motifs in random sequences and compare it to that observed in biological proteomes; the behaviour of random motifs is also studied. Most motifs exhibit a number of false positives significantly similar to the number of times they appear in randomized proteomes (=expected number of false positives). Interestingly, about 3% of the analysed motifs show a different kind of behaviour and appear in biological proteomes less than they do in random sequences. In some of these cases, a mechanism of evolutionary negative selection is apparent; this helps to prevent unwanted functionalities which could interfere with cellular mechanisms.

**Conclusion:**

Our thorough statistical and biological analysis showed that there are several mechanisms and evolutionary constraints both of which affect the appearance of functional motifs in protein sequences.

## Background

The detection of functional sequence patterns (motifs) in a yet uncharacterized protein is one of the most widely used and powerful methods for assigning a function to proteins in newly sequenced proteomes. Sequence patterns associated to functional motifs are usually generated manually in an attempt to maximize the number of sequences that clearly belong to the set functionally characterized by the motif (true positives, TP), while minimizing the number of unrelated sequences (false positives, FP) [1]. The possibility of estimating the number of false predictions is critical in evaluating the significance of finding a pattern in a protein sequence. The false positive rate of a pattern on a large protein database can be estimated from the number of matches expected to occur by chance [2,3]. In the following we will refer to this thesis as the 'classical hypothesis'. Both these authors measured the expectation of occurrence of PROSITE [4] functional patterns in a sequence database of size N, simply as the product of N and the amino acid probabilities in each position of the pattern. The individual amino acid probability values were calculated from the frequencies of residues in Swiss-Prot [5]. Sternberg [2] assumed the calculated expectations as a benchmark for evaluating motif matches on the Swiss-Prot database as annotated in PROSITE; Nevill-Manning and co-workers [3] used such expectations for assessing the specificity of motifs exhaustively generated from a multiple sequence alignment of related proteins. From this perspective, the number of occurrences of a motif in a set of proteins can be regarded as the sum of the functional occurrences plus the random occurrences, i.e. motif matches explained by the sequence composition alone [6].

The statistics of the number of occurrences of regular expression motifs in a random text, has been studied by several authors [[7-11] and related references]. Nicodème and co-workers [9] in particular, computed the theoretical expectation λ of the number of matches of PROSITE patterns on the PRODOM multi-alignment consensus sequences [12]; This analytical method uses computer science algorithms and combinatorial mathematics to give a constructive method for approximating (or calculating exactly, if the size of the problem allows for this) the distribution of number of matches of a given regular expression in a random text, the random text being modelled as either a Bernoulli sequence (independent letters with given probabilities of occurring) or a Markov sequence with given transition matrix. The matches can be defined as either (possibly) overlapping or as non-overlapping. For large texts, it is shown that the number of occurrences of the expression in the text will approximately follow a Normal distribution with mean and variance that can be calculated. In the application example, matching PROSITE motifs in a PRODOM based set of sequences, the whole database is treated as one large text and the letter frequencies used are thus computed as averages over all target sequences. The quantity λ was compared to the corresponding total number of observed matches O, with the finding that λ systematically underestimates O, a result that can be expected from the functional significance of PROSITE patterns.

Whereas events of over-representation of a motif are usually related to its biological significance, the analysis of the relationship between the expected number of random matches of a motif (λ) and its number of false predictions on a biological database (FP), might provide insight into the evolutionary nature of protein sequences. Indeed a chance match of a functional motif is unlikely to be functional wherever it is found in a protein: a motif will be recognized as functional only when occurring in the right conformation, location and cellular context. It is possible, however, that, at least in some cases, a match occurring for the "mere" sequence composition, fulfils physical conditions similar enough to those of the corresponding functional occurrence, thus giving rise to an "unwished" functionality. These cases would be, as a rule, disadvantageous for the cell and could be the object of a counter-selection process. On the other hand, a functional motif may involve such a high stringency of boundary conditions (protein conformation, cellular compartment, etc.) that its appearance (e.g. by exon shuffling or by a random mutation of a "pre-motif") in more than one unrelated protein family would be extremely unlikely to result in function interference.

The relationship between λ (expected) and FP (observed) has not been yet thoroughly investigated. In particular, a quantitative relationship, valuable for inferring the expected number of false positive matches for an uncharacterized pattern in a sequence database, is yet to be established. Neither has a qualitative analysis of the biological features of false occurrences, especially if such occurrences are associated to significantly higher or lower values of the corresponding theoretical expectations, been reported in the literature.

Here we propose a statistical analysis of the relationship between the theoretical expectation of the number of pattern matches in a database of random sequences and the number of false occurrences in a biological database. The study has been carried out for PROSITE patterns in the form of regular expression, but it is general enough to be applied to any motif of the same form.

Our approach is based on several concepts. First, we computed the expectation λ of a pattern P, as the mean number of hits in N database randomizations, instead of deriving it from some *a priori *statistical model for the occurrences of regular expressions (or motifs) in random texts. Second, a regression analysis on the set of *m *points (λ, FP), where *m *is the number of patterns under consideration, was used to determine an analytical relationship, which generalizes the (λ, FP) relationship to any motif and which can be, therefore, used for predictive purposes. Third, a study of statistical and biological features of the FP matches was performed for patterns displaying a number of false predictions in a biological database remarkably greater or lower than the expected number of hits in the corresponding randomized database. Our findings suggest diverse fascinating mechanisms and constraints occurring during evolution perhaps affecting the random appearance of functional motifs in protein sequences.

## Results

In the study of the relationship between the expected number of matches λ of a functional motif in a random dataset and the observed number of its false occurrences in the corresponding biological dataset, three important issues must be considered and discussed beforehand: the set of functional motifs used to carry out the experiments, the biological sequence dataset(s) in which the functional motifs are searched, and the statistical model adopted for establishing the theoretical expected number of random matches.

### The dataset of motifs

The set of functional motifs in the form of regular expressions was derived from the PROSITE database of patterns and generalized profiles, as described in methods.

Among other useful features, the PROSITE database provides, for each entry, complete lists of Swiss-Prot proteins manually verified for true positive (TP), false positive (FP), and false negative (FN) assignments [4].

Our work is dependent on the accuracy of PROSITE annotations of true and false positive matches. Random errors in the PROSITE annotation, however, should not affect the statistics of motif matches for two reasons: firstly, they are rare. In fact, true and false positives of PROSITE patterns are manually verified by expert curators through both the literature and the information retrieved from other databases such as Swiss-Prot or Pfam [13]. Secondly, random errors, by definition, do not have a preference for specific groups of patterns, and hence do not cause a systematic bias in our analysis. As it will be discussed, the presence of systematical errors, which may partly influence some results, has been evaluated and rejected.

### The sequence datasets

The complete Swiss-Prot sequence database is a general reference for motif statistics. However, it is redundant and its composition in protein families and organisms is still biased by the trends of the scientific community. The analysis carried out on human100 (see methods) must take into account that it is considerably smaller than the complete human proteome. The yeast proteome is only partially represented in the Swiss-Prot database. The two further proteomes analysed, *E. coli *and *M. jannaschii*, which instead are almost entirely represented in the Swiss-Prot database, belong to small organisms, and unfortunately the number of PROSITE patterns' FPs is too small for a solid statistical analysis (data not shown).

### The statistical model

The procedure adopted to estimate the expected number of random matches of a sequence motif does not rely on *a priori *assumptions about the statistical distribution of such matches (see methods). Moreover, it allowed us to overcome the obstacle of estimating the number of chance occurrences for those patterns characterized by variable spacers between more conserved positions. In fact, the problem of analytically calculating the probability of occurrence of this type of patterns in a random text has not yet been faced [11].

As a control, the analytical calculation of λ has also been performed by using the Nicodème and co-workers approach [9] for the subset of patterns with spacers of fixed length. The two approaches provide similar results, even if the Pearson correlation between λ, as calculated with our procedure, and FPs is always higher than the one between λ, as determined by Nicodème and co-workers, and FPs (see Table 1).

**Table 1:** Correlation and slope of PROSITE and *reversed *patterns

db	pattern	C1	C2	slope	R^2^
sprot	prosite	0.75	0.96	0.877 ± 0.019	0.71
	reversed	0.86	0.92	1.080 ± 0.023	0.71
human	prosite	0.84	0.88	0.908 ± 0.031	0.62
	reversed	0.86	0.89	1.055 ± 0.028	0.73
yeast	prosite	0.72	0.87	1.008 ± 0.046	0.58
	reversed	0.78	0.82	0.977 ± 0.049	0.54

(C1): Pearson correlation based on [9] (C2): Pearson correlation based on randomized datasets.

The Nicodème and co-workers approach is slightly different from the approach used in this work, since our method of randomizing by permutation within each sequence and of defining matches as simply absence or presence of the motif in the target sequences leads to a closer link between the actual sequence database and the randomized sequences and also to simpler statistical properties for the number of matches. The major consequence concerns patterns abundant in cysteines. Since proteins are particularly heterogeneous regarding the density of the cysteines, the value of λ of some cysteine-rich patterns turned out to be underestimated by Nicodème and co-workers and overestimated by our procedure. Such patterns were discarded from the regression analysis.

**Figure 1:**
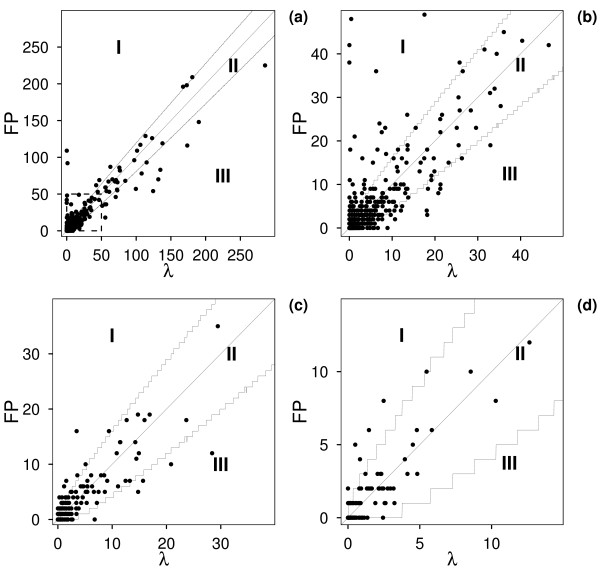
**The number of false positives *versus *the number of random matches**. The mean number of matches of a pattern in N (N = 1000) outcomes of a biological sequence dataset randomization (λ) *versus *the number of false positives (FP) on the biological dataset. Each point corresponds to a PROSITE pattern. The two non-straight lines over and below the bisector delimitate the 95% confidence intervals around the line λ = FP and divide the plan into three regions: I, II and III. Fig. 1a and b (which is a zoom of the square area of Fig. 1a) display sprot100 data, whereas Fig. 1c and d represent human100 and yeast100 data, respectively.

Fig. 1 shows the plot (λ, FP), where λ is the mean number of matches of a pattern in N (N = 1000) outcomes of a biological sequence dataset randomization, and FP is the number of false positive matches in the original dataset. The plot displays a linear behaviour, and the Pearson correlation between the x-axis and y-axis values is > 0.87 for all the sequence datasets considered (Table 1). A regression line was then fitted to the data. The values of the slope are reported in Table 1 for each sequence dataset. It is worth noting that the slope is lower than one for both sprot100 and human100, whereas it is one for yeast100, within the evaluated error.

### PROSITE and reversed patterns statistical analysis

The lines defining the 95% confidence interval divide the (λ, FP)-plane into three regions (see Fig. 1). Patterns belonging to the first region (I) are such that FP > λ_R _(*outliers *with observed number of random matches greater than the corresponding expected number). Patterns with λ_L _< FP < λ_R _are in the central region (II), and those belonging to the third region (III) are such that FP < λ_L _(*outliers *with observed number of random matches lower than the corresponding expected number).

The number and features of patterns from regions I, II and III were analysed separately and then compared. The majority of the patterns belong to the central region (II), while few patterns have a number of FPs significantly greater than expected by chance (region I) in every dataset considered. sprot100 and human100 also display a subset of outliers which have a number of FPs significantly lower than expected by chance (region III).

On a biological dataset, matches of random patterns, i.e. derived from differently ordered positions of functional motifs, probably occur by chance. We obtained randomized PROSITE patterns by reversing their regular expressions (see methods). This procedure should generate patterns with no functional significance. Being made up of the same composition of residues as the original PROSITE patterns, these random patterns display the same statistical properties, including the information content [14], as the original ones. The statistical analysis of the *reversed *patterns revealed a high correlation between matches in the random datasets and matches in the biological datasets and a regression slope that is always equal to one (Table 1).

The comparison between the slope values of PROSITE and *reversed *patterns shows that, whereas *reversed *patterns have slope ≈ 1 on every datasets considered (i.e. λ ≈ FP), PROSITE patterns display a slope value lower than one, namely, on average, a higher number of matches in the random datasets as opposed to the biological datasets in the cases of sprot100 and human100.

### PROSITE patterns

In the following, the detailed analysis performed on the sprot100 dataset is reported.

#### Patterns in region II

1196 PROSITE patterns lay in region II (see Table 2). Patterns where FP = 0 are predominant in this region. The fact that the majority of patterns fall in this region shows that our model confirms the "classical hypothesis" [2,3], although the slope < 1 (see table 1) indicates a general tendency of having FP < λ.

**Table 2:** The information content (IC)

Pattern region		N	IC average	Q_25_	Q_75_
I	sprot100	63 (5%)	26.8 ± 0.7	23.3	30.7
III		36 (3%)	20.3 ± 0.5	18.9	21.9
II		1196 (92%)	33.3 ± 0.3	26.4	37.4
II*		333	24.2 ± 0.2	22.3	26.3
μ = FP = 0		863	36.9 ± 0.4	30.2	40.5
					
I	human100	20	24.5 ± 1.2	20.6	28.7
III		2	17.3 ± 0.5	17.1	17.5
II		830	32.8 ± 0.4	25.2	37.4
II*		150	21.9 ± 0.2	20.3	23.6
μ = FP = 0		680	35.2 ± 0.4	28.3	39.0
					
I	yeast100	10	24.4 ± 2.1	21.4	29.2
III		0	-	-	-
II		598	31.9 ± 0.4	25.4	35.6
II*		65	20.8 ± 0.3	19.5	21.8
μ = FP = 0		532	33.2 ± 0.4	26.9	36.5

Region II* is the region II without patterns for which λ = FP = 0; Q_25 _is the 25^th ^percentile and Q_75 _is the 75^th ^percentile; N is the number of patterns in the corresponding region.

Even though the majority of patterns has a number of FPs similar to the number of matches expected by chance, we need to take into account that the cell machinery is constantly subject to mutational events and, consequently, to the force of natural selection. It is therefore reasonable that, on average, regarding the accidental occurrence of functional motifs, protein sequences do not behave as mere aggregations of letters. Thus, we hypothesize that, albeit if as a very small effect, the tendency of having FP < λ might reflect the evolutionary pressure (non-uniformly distributed) against the random appearance of functional motif matches. This hypothesis of "mild counter-selection" cannot be proved. It is, however, strengthened by the fact that, for non-functional patterns, we found FP ~λ (see below).

#### Outliers of region I

Sixty-three PROSITE patterns lay in region I (Table 2 and 3). In a Poisson distribution (see Methods) the number of patterns expected to fall by chance in this region is 18 (Table 4). To investigate possible reasons why these motifs display a much higher number of false positives than expected by chance, we analyzed those patterns in some detail and identified two groups:

**Table 3:** The order propensity (OP) value (GlobPlot)

Pattern region	dataset	N	OP average	Q_25_	Q_75_
I	sprot100	63	0.37 ± 0.03	0.18	0.50
III		31	0.25 ± 0.03	0.14	0.33
II*		252	0.31 ± 0.02	0.13	0.43
					
I	human100	19^1^	0.25 ± 0.06	0.03	0.30
III		2	0.14 ± 0.02	0.14	0.16
II*		116^1^	0.22 ± 0.02	0.00	0.30
					
I	yeast100	10	0.41 ± 0.08	0.23	0.49
III		0	-	-	-
II*		53^1^	0.32 ± 0.04	0.17	0.39

N is the number of patterns. The number of patterns in region III and region II* differs from the number of patterns in the corresponding regions of table 2 because only for patterns with FP > 0 the OP value can be calculated.

**Table 4:** Number of expected by chance and observed PROSITE patterns in regions I and III

		95%				99%			
		
		I	p-value	III	p-value	I	p-value	III	p-value
sprot100	exp	18	<0.0001	3	<0.0001	4	<0.0001	0.5	<0.0001
	obs	63		36		54		22	
human100	exp	9	0.0012	0.6	n.s.	2	0.0002	0.1	0.0002
	obs	20		2		9		3	
yeast100	exp	5	0.0385	0.1	n.s.	1	<0.000	0	n.s.
	obs	10		0		7	1	0	

p-value is the p-value assigned to the number of outliers observed (obs) *versus *the number expected (exp) from the Poisson distribution and n.s. stands for p-value > 0.05.

1) patterns that are mis-annotated in PROSITE (if correctly annotated they would belong to region II);

2) true outliers.

1) mis-annotated patterns are examples of patterns whose occurrences, annotated as FPs for a specific PROSITE pattern, are able to identify functionally or structurally relevant regions in protein families different from the true positives protein family but anyway functionally related to it. Two of them are PS00103 and PS00120.

The majority of PS00103 true positives are ribose-phosphate pyrophosphokinases, which contain the Pribosyltran Pfam [15] domain. The PS00103 motif is well conserved in this domain. Nearly all the false positives are adenine phosphoribosyltransferase (APRT), which also have a Pribosyltran Pfam domain. It would be, therefore, more appropriate to use this motif to describe the wider family of Pribosyltran domain containing proteins.

PS00120 is the PROSITE motif built around the serine active site of lipases. Many false positives are found among esterases. This pattern is very conserved (especially the stretch around the catalytic site) in the Pfam alpha/beta hydrolase fold domain, which is found in several protein families including lipases and esterases.

Another interesting example of mis-annotated pattern is PS00675 which is an atypical ATP-binding motif A (p-loop) characteristic of bacterial regulatory proteins involved in the ATP-dependent interaction with sigma-54 [16,17], such as algB, dcdT, flbD, hoxA, hupR1, hydG, ntrC, pgtA or pilR. The majority of false positives of this motif belong to the eukaryotic rab-like ypt1 family of GTP-binding proteins. Thus, PS00675 might be either an ancestral motif originally duplicated by mechanisms such as exon shuffling, or a case of convergent evolution. In both cases, at present, it co-exists in bacterial and eukaryotic proteins with no functional interference.

2) The number of true outliers is in agreement to what expected by chance from the Poisson distribution. One interesting example of a true outlier among many others is represented by PS00014, which is annotated in the ELM database (Eukaryotic Linear Motif. database) [18,19] as TRG_ER_KDEL. This is a short C-terminal signal motif which is strictly conserved in the major endoplasmic reticulum (ER) proteins, and which allows proteins that permanently reside in the ER lumen to be distinguished from newly synthesized secretory proteins. In sprot100, this motif is also detected in 53 non-related proteins, which are clearly not located in the ER (because they are of bacterial or viral origin, for example).

A further analysis was performed on the PS00014 false positive sequences annotated in the Swiss-Prot release 50.1. The subcellular location of each protein was retrieved by means of the Swiss-Prot annotation. Out of the 100 FPs, only two reside in the ER: one is annotated in the manually curated ELM database as true positive and the other could potentially belong to the set of true positives.

We hypothesize that a "proliferation" (e.g. by gene duplication) of FPs might have occurred, in some cases, in organisms or cellular compartments where their occurrence does not interfere with the function associated to the motif. In fact, it is unlikely that negative selection will act on accidental duplication events if these do not perturb the cell functionality.

#### Outliers of region III

Thirty-six patterns lay in region III (Table 2 and 3). Following the Poisson distribution, the number of patterns expected to fall in this region by mere chance is 3 (see Table 4). These patterns have a number of FPs which is lower than λ. It should be also noticed that they display low information content and their FPs occur in more disordered (more exposed) regions of proteins (see next section). Our findings suggest that they are likely to result in "unwished" functionalities and, therefore, are good candidates for negative selection.

There are two possible arguments that might be at variance with the negative selection hypothesis: The number of FPs is underestimated because of

1) a systematic underestimate in the PROSITE annotation for true positives

2) a non-complete coverage of the sequence datasets

Here we discuss both arguments and explain why neither of them hold.

1) Some proteins might have been erroneously identified as true positives by similarity alone, indeed. If this were the case, we would expect the real number of false positives to be higher and these patterns to belong to region II. In other words, for these patterns, the number of true positives as assigned in PROSITE (TP) would consist of a number of experimental true positives plus a number of mis-annotated true positives. In such a case we would expect to find that the difference between the number of matches expected by chance and the number of PROSITE false positives is correlated to TP. We found that this correlation is < 0.2, and rejected the hypothesis of a systematic error in the annotation of patterns in region III.

2) It could be proposed that, by increasing the number of sequences in the datasets, the number of FPs might grow more rapidly than the number of random matches, resulting in a shift of patterns from region III to region II.

In order to verify this possibility, we started from non-redundant versions of the datasets under study and increased progressively their redundancy (this corresponds to augmenting the number of sequences). Then we studied the relationship between FP and λ and found that the number of FPs increases more slowly than the number of random matches in the dataset (data not shown). This result implies that the addition of sequences to a dataset would either relocate some patterns of region II in region III or leave the situation unaffected.

### Information content and disorder propensity

The patterns' mean information content (IC) was calculated as explained in Methods [14]. The IC of a pattern is directly proportional to the length of the pattern and inversely proportional to its degeneracy. Patterns with high IC are in general longer and display lower degeneracy than patterns with low IC. This implies that a high IC is advantageous for the selectivity of a motif. We found that the highest IC value is attained by patterns for which λ = FP = 0 (see Table 2).

Interestingly, for each sequence dataset considered the mean IC decreases from patterns belonging to region I to those of region III. In particular, a single pattern analysis showed that the vast majority of patterns in region III are short (few positions) and non-degenerate (few amino acid types permitted in each position). This means that these patterns are less subject to conformational constraints.

Patterns were compared also for the propensity of their false positive matches to occur in ordered/disordered protein regions (see Methods). Ordered regions typically contain regular secondary structures packed into a compact globule [20]. Ordered regions, therefore, tend to be more buried, and therefore more hidden, than disordered ones. On average, false positive matches of patterns belonging to region III have a higher propensity of occurrence in protein disordered segments than those belonging to region I and II have (Table 3). These findings suggest that the patterns of group III tend to occur in more exposed and less structurally constrained regions of proteins, as compared to patterns of groups I and II and especially to those for which λ = FP = 0. The evolutionary implications of these results will be further addressed in the 'Discussion' section.

### Reversed patterns

The most interesting result is that these patterns display a regression slope which is always equal to one (Table 1). A detailed analysis was performed in order to establish if all the reversed patterns are non-functional. 352 reversed patterns matching PROSITE patterns were removed (see Methods). The great majority of the remaining patterns lies in the 95% confidence interval. Few of them fall in region I (thirty-eight patterns for sprot100, eleven for human100 and six for yeast100). These are predominantly patterns highly conserved in some Pfam [15] family or domain (22 for sprot100, 7 for human100 and 4 for yeast100). For example, the reversed of PS00850 is found almost solely in regulator of ribonuclease activity A proteins and, moreover, it is conserved in the demethylmenaquinone methyltransferase Pfam domain present in these proteins. Hence, these are clearly non-random patterns that cannot be considered as non-functional and are, therefore, excluded from the regression analysis.

Finally, in region I, only fourteen patterns for sprot100, four for human100 and two for yeast100 are apparently non-functional. These numbers are in agreement with what it is expected by chance in this region (12 for sprot100, 5 for human100 and 3 for yeast100).

There is also a subset of reversed patterns falling in region III (twenty-five patterns for sprot100, four for human 100 and none for yeast). These patterns are less frequent (and in seven cases in sprot100 even much less frequent) in the biological database than in the random database. This observation together with an ad hoc analysis of the patterns, suggests that some of them share a particular amino acid compositions whose random appearance in a protein would have a high probability of interfering with the normal cell functionality. In this case it is possible that they are functional and conceivable that could be subjected to events of negative selection.

## Discussion

The first telling conclusion is that the great majority of functional motifs have a number of false occurrences comparable to the number of matches on a random database (i.e. they belong to the region II of the (λ, FP)-plane).

This result is in agreement with works such as [2] and [3]. Thus, it seems that, in most cases, proteins are not affected if a stretch of amino acids, selected during evolution and carrying a structural or functional ability, appears in a proteome only by chance. In other words false occurrences of a functional motif do not generally compete with true positive ones. Indeed, there are several boundary conditions a motif has to fulfil in order to be functional in a protein or to be recognized by interactors. In the majority of cases, interactors are specific enough to discriminate between an exceedingly high number of similar sites and avoid mis-functionalities. On the other hand, sites that are similar to target motifs with limited specificities can be found not to interfere since they are hidden or appear in other cellular contexts, different cell types or tissues or even different organisms.

This possibility is in agreement with the order/disorder propensity data for false positive matches of patterns belonging to region I and II, i.e. patterns for which the number of false positives is higher or similar to the expected number of random hits (Table 3). In fact, we observed that false positive matches of patterns in region I and II are, on average, in more ordered areas than those of patterns in region III, suggesting how proteins may avoid cases of mis-recognition by interactors with little specificity.

A careful analysis of patterns for which the number of FP is notably greater than the number of matches expected by chance revealed several interesting features. First, some cases of mis-annotation have been identified. Besides these cases, which are only relevant for the quality of a pattern database, we observed several patterns whose false positive matches are functionally important in proteins non-related to and not co-localizing with proteins matching the true positives. In some other cases it seems that false positive matches proliferate, even with no functional relevance, in proteins spatially segregated from true positives. In both situations, evolution has been apparently more permissive or even rewarding towards compositions of amino acids belonging to non-interfering proteins.

Another solution to the problem of functional pattern specificity might be that, during evolution, longer and not too variable functional modules have been rewarded [21]. In this case, not only could the true positive site be unique, but there might not be any sites with one mismatch. In this regard, it is worth noting that between 72% (in sprot100) and 89% (in yeast100) of patterns are such that λ = FP = 0 and that the mean information content (IC) of these patterns is much higher than the mean IC of any other group considered.

What can be said in terms of evolution about patterns of group III? These are patterns whose number of false positives is lower than expected by chance. They may belong to group III for three principal reasons.

The first one might be a systematic error in the PROSITE annotation for true positives, something which has been excluded by our analysis.

The second reason concerns the randomization model. We have seen that, at least in some cases, the expected number of chance matches of patterns rich in cysteines, depends on the statistical model adopted to evaluate it. Thus, one can imagine that patterns belonging to group III have some particular features, thus making their rate of occurrence in a random dataset dependent on the procedure adopted to randomize the dataset. An accurate analysis of the patterns, however, did not reveal any distinctive features supporting this hypothesis.

The third reason has evolutionary implications. The patterns of group III are characterized by a preponderance of residues predisposed to be present in more disordered, i.e. more exposed, regions of proteins. Moreover, short patterns, with little IC, undergo less conformational constraints than long patterns, with high IC. Thus, at least two important conditions of functionality are more easily fulfilled by this group of patterns.

If a false occurrence of a functional motif results in an "unwished" functionality, the protein(s) carrying it would be rejected during evolution. Cases of evolutionary negative selection have been recognized in immunology [22] and are likely to have an important role in many biological systems [23,24]. Zarrinpar and co-workers [25] hypothesized negative selection against non-specific interactions as a mechanism for specificity enhancement of a particular SH3-ligand pair in yeast. Discrimination against proteins holding sequence *consensi *that are, for example, possible competitors in recognition or interaction processes, might account for the observed non-random distribution of false positive matches of some patterns. For example, a false positive match of a SH3 ligand consensus might result in promiscuous SH3 binding in the case of subcellular co-localization and temporal overlapping with a true occurrence of the ligand peptide.

The lower IC and the observed disorder tendency of the false positives of patterns of group III, compared to patterns of groups I and II, support the hypothesis of their counter-selection.

Evolutionary negative selection, however, is difficult to prove because so few biological cases have been clearly identified and there is only indirect information on the "products" of such a mechanism. Furthermore, organisms have developed several strategies for avoiding protein mis-functionalities, which may or may not operate through the mechanism of negative selection. Finally, counter-selection is more likely to occur in complex organisms, where evolution deals with duplication and mutation of a greater number of genes or portions of them. These organisms, however, are still insufficiently represented in the Swiss-Prot databank. More data will be available to support or disprove our hypotheses as soon as large and fully-annotated proteomes become available.

## Conclusion

We presented a general numerical analysis exploring the different mechanisms used by Nature in order to prevent random occurrences of a functional motif interfering with the proper functioning of a living cell.

We used quantitative parameters (information content, disorder) to analyse PROSITE patterns sharing a common behaviour in terms of their appearance in random sequences. This analysis, together with a further literature study of the single motifs, led to the conclusion that, in the majority of the cases (>90%), FPs of functional motifs are not counter-selected during evolution: Apparently, the probability that a false positive occurs in the same organism, tissue, cell compartment, and protein region of a true positive, is low enough to prevent the risk of functional interference.

Our results therefore show that, for the majority of proteins, the mere presence of a motif is not enough to entail a particular function and therefore additional constraints must be satisfied. Furthermore, we identified a subgroup of motifs whose false positives have been subjected to events of counter-selection.

## Methods

### Datasets

A set of functional patterns, in the form of regular expressions, was extracted from the complete PROSITE database [4] release 28.27, discarding patterns that are so general that PROSITE cannot classify true and false matches, and patterns restricted to either the N- or C-terminal of a sequence. This filtering procedure produced a set of 1295 functional motifs whose true (TP) and false positive (FP) sequences were identified from the PROSITE annotations and cross-referenced on the Swiss-Prot sequence database [5], release 42.

In the following, we will refer to "matches", as the number of sequences with at least one match, i.e. we did not consider multiple instances of a pattern in the same sequence.

The analysis described in this work was performed on five sequence datasets: the complete Swiss-Prot database after the removal of sequences containing one or more undetermined amino acid (sprot100, 145203 sequences), the set of 10632 *H. sapiens *sequences (human100), and the set of 4925 *S. cerevisiae *sequences (yeast100) derived from sprot100. The two further subsets analysed, are *E. coli *(complete proteome 4,338 proteins) and *M. jannaschii *(1,782 proteins), which are almost entirely represented in the Swiss-Prot database. Notice that the entire human and yeast proteomes comprise 35,093 proteins and 6,224 proteins, respectively. For each sequence dataset, the group of PROSITE patterns displaying at least one true positive hit was identified: 1295 patterns for sprot100, 855 for human100, 607 for yeast100, 639 for *E. coli *and 260 for *M. jannaschii*.

Data on the complete proteomes size are extracted from the Integr8 EMBL-EBI genome statistics [26]. Sequence and pattern datasets are available on request.

### Sequence databases randomization

In order to preserve the local sequence composition and to account for finite sequence length effects, the sequence databases were randomized by reshuffling each single sequence. We define a shuffled sequence of a sequence S as one in which the number of each kind of amino acid is *exactly *the same as in S [27]. The randomization program was written in C using the GNU scientific library for random number generation and sequence shuffling [28].

Each dataset under consideration was randomized N (N = 1000) times. The randomization program is available on request.

### Statistical model

The basic dataset was randomized N times and the number of matches with all selected patterns was counted for each randomization. For each pattern, the average number of matches was then calculated. The probability distribution of matches of a given pattern was assumed to be Poisson [29]. This assumption was checked by plotting average number of matches for each pattern against the corresponding variance, finding a satisfactorily linear plot with unit slope (as expected in a Poisson setting).

More precisely, our randomization and match counting method implies that the random variable which counts the number of matches of a given motif in the randomized database will be the sum of a very large number (the number of sequences in the database) of Bernoulli variables (0/1 variables) with different but always small probabilities for the value 1, representing the presence of the motif in a given, permuted, single sequence. The sum of these probabilities is the expected number of matches of the motif in the whole database. The distribution of the number of matches can be approximated by the Poisson distribution regardless of the size of the expected number of matches as long as the largest single probabilities discussed above are small.

For large expected values, the Poisson distribution will simply be approximately Gaussian. In any case, the variance of the number of matches will always be smaller, although usually almost equal to, the variance implied by the Poisson distribution. Thus, use of the Poisson distribution will be conservative. In other words, considering a given observation as significantly deviating from an expected distribution will be more difficult under the Poisson distribution than under the "exact" distribution.

The number N of randomizations was set at 1000, yielding a relative error less than 3% in the determination of average numbers of matches larger than 1 and, although relative error may be larger, yielding an even smaller absolute error for averages smaller than 1.

We then constructed an exact confidence interval (c.i.) comprising at least 95% probability for each value λ of the observed average numbers of matches by iteratively summing probabilities p around λ (with probability values taken from the theoretical Poisson distribution) until Σp ≥ 0.95. The values of λ and the c.i. are plotted in Figure 1. Since the Poisson distribution is discrete, the calculation produced irregular lines, which indicate the upper and lower limits [λ_L_, λ_R_] of the prediction intervals and divide the (λ, FP)-plane into three regions (I, II and III). Furthermore, for each observed value of λ (expected number of matches under randomization), the corresponding value of effectively observed FP is used to generate a point in the figure. Points lying outside the c.i. can be considered as candidates for "extra-random effects".

It should be noticed that the discrete nature of the Poisson distribution implies that the probability above and below each c.i. will not be exactly 2.5% and may be different for each λ. This affects the expected number of patterns to be found outside the c.i. "by pure chance". However, one can calculate the exact expected number of patterns lying below or above the prediction interval by simply summing the probabilities corresponding to values left out from the intervals (There are good theoretical grounds for assuming that also the number of "chance outliers" follows the Poisson distribution).

The above construction of prediction intervals and relative calculation of "outlier patterns" was repeated at the 99% probability level, in addition at the 95% level in the previous experiment. In both cases, we found a number of outlier patterns lying above (region I) or below (region II) the prediction intervals significantly higher than expected by chance (see Table 4).

### Regression model

All the statistical computing was performed using the R language and environment [30].

For each pattern, λ was plotted *versus *the PROSITE pattern false positive number of matches and a line was fitted to the data, i.e. a regression analysis was applied to the set of points (λ, FP).

Given that false positive matches are expected to occur by chance alone in a biological database, it is reasonable to assume that the response variable, FP, comes from a Poisson distribution. From this assumption it follows that the variance of the variable FP increases with its mean value: to correct for the heteroskedasticity of residuals we defined a vector of weights (w) for k observations as w = (1/(λ_1 _+ 1),...,1/(λ_i _+ 1),...,1/(λ_k _+ 1)). In other words, the influence of an observation on the fit decreases as its value increases.

From the equation for the line of best fit, the slope and intercept values were then calculated.

### Information content

PROSITE patterns are written in the form:

*P *= *A*_1 _- *x*(*i*_1_, *j*_1_) - *A*_2 _- *x*(*i*_2_, *j*_2_) -...- *x*(*i*_*p*-1_, *j*_*p*-1_) - *A*_*p *_where A_i _is a non-empty set of amino acid symbols and i_k _≤ j_k_, which are integers, indicate the length of a wildcard x. We define the information content (IC) of a PROSITE type pattern as ([31,14]):

I(P)=∑i=1pI1(Ai)−c×∑k=1p−1(jk−ik)
 MathType@MTEF@5@5@+=feaafiart1ev1aaatCvAUfKttLearuWrP9MDH5MBPbIqV92AaeXatLxBI9gBaebbnrfifHhDYfgasaacH8akY=wiFfYdH8Gipec8Eeeu0xXdbba9frFj0=OqFfea0dXdd9vqai=hGuQ8kuc9pgc9s8qqaq=dirpe0xb9q8qiLsFr0=vr0=vr0dc8meaabaqaciaacaGaaeqabaqabeGadaaakeaacqWGjbqscqGGOaakcqWGqbaucqGGPaqkcqGH9aqpdaaeWbqaaiabdMeajnaaBaaaleaacqaIXaqmaeqaaOWaaeWaaeaacqWGbbqqdaWgaaWcbaGaemyAaKgabeaaaOGaayjkaiaawMcaaaWcbaGaemyAaKMaeyypa0JaeGymaedabaGaemiCaahaniabggHiLdGccqGHsislcqWGJbWycqGHxdaTdaaeWbqaamaabmaabaGaemOAaO2aaSbaaSqaaiabdUgaRbqabaGccqGHsislcqWGPbqAdaWgaaWcbaGaem4AaSgabeaaaOGaayjkaiaawMcaaaWcbaGaem4AaSMaeyypa0JaeGymaedabaGaemiCaaNaeyOeI0IaeGymaedaniabggHiLdaaaa@549C@

where *c *is a constant (normally *c *= 0.5) and I_1_(A_i_) is the information content of a single position A_i_:

I1(Ai)=−∑a∈Σ(pa×log⁡2(pa))+∑a∈Ai(papAi×log⁡2(papAi))
 MathType@MTEF@5@5@+=feaafiart1ev1aaatCvAUfKttLearuWrP9MDH5MBPbIqV92AaeXatLxBI9gBaebbnrfifHhDYfgasaacH8akY=wiFfYdH8Gipec8Eeeu0xXdbba9frFj0=OqFfea0dXdd9vqai=hGuQ8kuc9pgc9s8qqaq=dirpe0xb9q8qiLsFr0=vr0=vr0dc8meaabaqaciaacaGaaeqabaqabeGadaaakeaacqWGjbqsdaWgaaWcbaGaeGymaedabeaakmaabmaabaGaemyqae0aaSbaaSqaaiabdMgaPbqabaaakiaawIcacaGLPaaacqGH9aqpcqGHsisldaaeqbqaamaabmaabaGaemiCaa3aaSbaaSqaaiabdggaHbqabaGccqGHxdaTcyGGSbaBcqGGVbWBcqGGNbWzdaWgaaWcbaGaeGOmaidabeaakmaabmaabaGaemiCaa3aaSbaaSqaaiabdggaHbqabaaakiaawIcacaGLPaaaaiaawIcacaGLPaaaaSqaaiabdggaHjabgIGiolabfo6atbqab0GaeyyeIuoakiabgUcaRmaaqafabaWaaeWaaeaadaWcaaqaaiabdchaWnaaBaaaleaacqWGHbqyaeqaaaGcbaGaemiCaa3aaSbaaSqaaiabdgeabnaaBaaameaacqWGPbqAaeqaaaWcbeaaaaGccqGHxdaTcyGGSbaBcqGGVbWBcqGGNbWzdaWgaaWcbaGaeGOmaidabeaakmaabmaabaWaaSaaaeaacqWGWbaCdaWgaaWcbaGaemyyaegabeaaaOqaaiabdchaWnaaBaaaleaacqWGbbqqdaWgaaadbaGaemyAaKgabeaaaSqabaaaaaGccaGLOaGaayzkaaaacaGLOaGaayzkaaaaleaacqWGHbqycqGHiiIZcqWGbbqqdaWgaaadbaGaemyAaKgabeaaaSqab0GaeyyeIuoaaaa@6D17@

*p*_a _is the probability (calculated from the frequency of amino acid *a *in the dataset under consideration) of amino acid *a*, Σ is the set of all amino acids and PAi=∑a∈Aipa
 MathType@MTEF@5@5@+=feaafiart1ev1aaatCvAUfKttLearuWrP9MDH5MBPbIqV92AaeXatLxBI9gBaebbnrfifHhDYfgasaacH8akY=wiFfYdH8Gipec8Eeeu0xXdbba9frFj0=OqFfea0dXdd9vqai=hGuQ8kuc9pgc9s8qqaq=dirpe0xb9q8qiLsFr0=vr0=vr0dc8meaabaqaciaacaGaaeqabaqabeGadaaakeaacqWGqbaudaWgaaWcbaGaemyqae0aaSbaaWqaaiabdMgaPbqabaaaleqaaOGaeyypa0ZaaabeaeaacqWGWbaCdaWgaaWcbaGaemyyaegabeaaaeaacqWGHbqycqGHiiIZcqWGbbqqdaWgaaadbaGaemyAaKgabeaaaSqab0GaeyyeIuoaaaa@3BD4@.

This measure is based on Shannon's theory [32] and takes also into account the increase in uncertainty due to variable spacers of patterns.

### The control: reversed patterns

As a control, the statistical analysis was also performed on randomized (i.e. non functional) PROSITE patterns. There are too many possible permutations when PROSITE patterns are used, thus we chose to reverse the pattern positions as a randomization procedure.

The *expected *number of matches in random datasets and the *observed *number of matches in the corresponding biological datasets were calculated for the *reversed patterns*, and a regression analysis was carried out. The (λ, FP) pairs of values for PROSITE patterns of each region (I, II, and III) were compared to the (λ, O) pairs of values for the *reversed patterns*, where λ is once again the arithmetical mean of the number of matches on N (N = 1000) dataset randomizations and O is the observed number of hits on the corresponding biological dataset.

352 reversed patterns were found to match a PROSITE pattern. These were considered functional and therefore discarded. It is possible, however, that some of the remaining reversed patterns still encode a function. This is difficult to detect by means of an automated procedure. Thus, we performed a manual analysis of the reversed patterns displaying an *observed *number of matches very different from the number of *expected *matches (regions I and III). Reversed patterns matching conserved regions of some Pfam [15] domains were also discarded. The regression analysis was carried out, for each sequence dataset, on the reversed patterns retained after the filtering procedure only.

### The false positives "disorder"

Protein disorder is described as the lack of regular secondary structure and a high degree of flexibility in the polypeptide chain [33]. Several computational methods exist for identifying disordered residues within proteins [34]. To infer the tendency of PROSITE patterns false positive hits of being in either disordered or ordered/globular regions of proteins, we applied GlobPlot [20], a predictor of protein disorder and globularity, to the sequences of the datasets considered.

GlobPlot is based on propensities, *P*, for all amino acids to be in globular or non-globular states. The leading hypothesis is that the tendency for disorder of an amino acid *a *can be expressed as *P*(*a*) = *RC*-*SS *where *RC *and *SS *are calculated as the frequencies for the amino acids' appearing either in regular secondary structures (helices or strands) as defined by DSSP [35] or outside them ('random coil', loops, turns etc.). Given a protein sequence of length L, a sum function is defined as:

Dis(ai)=∑j=1iP(aj)for i=1,...,L
 MathType@MTEF@5@5@+=feaafiart1ev1aaatCvAUfKttLearuWrP9MDH5MBPbIqV92AaeXatLxBI9gBaebbnrfifHhDYfgasaacH8akY=wiFfYdH8Gipec8Eeeu0xXdbba9frFj0=OqFfea0dXdd9vqai=hGuQ8kuc9pgc9s8qqaq=dirpe0xb9q8qiLsFr0=vr0=vr0dc8meaabaqaciaacaGaaeqabaqabeGadaaakeaafaqabeqacaaabaGaemiraqKaemyAaKMaem4Cam3aaeWaaeaacqWGHbqydaWgaaWcbaGaemyAaKgabeaaaOGaayjkaiaawMcaaiabg2da9maaqahabaGaemiuaa1aaeWaaeaacqWGHbqydaWgaaWcbaGaemOAaOgabeaaaOGaayjkaiaawMcaaaWcbaGaemOAaOMaeyypa0JaeGymaedabaGaemyAaKganiabggHiLdaakeaacqqGMbGzcqqGVbWBcqqGYbGCcqqGGaaicqWGPbqAcqGH9aqpcqaIXaqmcqGGSaalcqGGUaGlcqGGUaGlcqGGUaGlcqGGSaalcqWGmbataaaaaa@5051@

where *P*(α_i_) is the propensity for the *i*th amino acid. The sum function defines a curve, which provides the regions of disorder/order along the sequence.

We then identified the position along the sequences of the false positive matches. The order propensity of a pattern (OP ∈ [0,1]) was defined as the ratio of residues of the pattern belonging to an ordered region (as defined in [20]) to the total number of residues of the pattern. The lower the value of OP of a pattern, the higher its propensity of lying in disordered regions.

## Abbreviations

TP, true positive; FP, false positive; FN, false negative; O, observed; IC, information content; OP, order propensity.

## Authors' contributions

AV conceived the study and participated in its coordination, performed the data analysis and authored the manuscript. PFG carried out the statistical study, produced the data and contributed to the manuscript authoring and revision. EF played a crucial role in the design of the study, in the analysis and interpretation of data and in the manuscript authoring. GST helped in the design of the statistical strategy and assessed its robustness. GST authored the statistics sections. GA took part in the analysis and interpretation of data. MHC participated in the design and supervision of the research. MHC revised the manuscript and gave final approval of the version to be published. All authors read and approved the final manuscript.
